# Quality of Service Aware Orchestration for Cloud–Edge Continuum Applications

**DOI:** 10.3390/s22051755

**Published:** 2022-02-23

**Authors:** Adrián Orive, Aitor Agirre, Hong-Linh Truong, Isabel Sarachaga, Marga Marcos

**Affiliations:** 1 Departamento de Ingeniería de Sistemas y Automática, University of the Basque Country, 48013 Bilbao, Spain; isabel.sarachaga@ehu.es (I.S.); marga.marcos@ehu.es (M.M.); 2 Ikerlan, 20500 Mondragon, Spain; aagirre@ikerlan.es; 3 Department of Computer Science, Aalto University, 02150 Espoo, Finland; linh.truong@aalto.fi

**Keywords:** orchestration, Cloud–Edge continuum, Edge computing, fog computing, quality of service

## Abstract

The fast growth in the amount of connected devices with computing capabilities in the past years has enabled the emergence of a new computing layer at the Edge. Despite being resource-constrained if compared with cloud servers, they offer lower latencies than those achievable by Cloud computing. The combination of both Cloud and Edge computing paradigms can provide a suitable infrastructure for complex applications’ quality of service requirements that cannot easily be achieved with either of these paradigms alone. These requirements can be very different for each application, from achieving time sensitivity or assuring data privacy to storing and processing large amounts of data. Therefore, orchestrating these applications in the Cloud–Edge computing raises new challenges that need to be solved in order to fully take advantage of this layered infrastructure. This paper proposes an architecture that enables the dynamic orchestration of applications in the Cloud–Edge continuum. It focuses on the application’s quality of service by providing the scheduler with input that is commonly used by modern scheduling algorithms. The architecture uses a distributed scheduling approach that can be customized in a per-application basis, which ensures that it can scale properly even in setups with high number of nodes and complex scheduling algorithms. This architecture has been implemented on top of Kubernetes and evaluated in order to asses its viability to enable more complex scheduling algorithms that take into account the quality of service of applications.

## 1. Introduction

In the last decade, the Cloud computing paradigm has improved the computing capabilities of business applications in many domains while decreasing the total cost of ownership. Typical Cloud computing clusters are made of powerful devices located in big datacenters, under proper temperature conditions, and with stable network and energy supplies. Virtualization technologies empowering these devices enable third companies to pay for the computing resources as they need them, instead of hosting their own infrastructure [1].

Currently, Cloud applications consist of lightweight virtualization components that are deployed as a set of containers, improving space requirements and deployment times compared to virtual machines [2]. These containers are managed by Container Orchestration Engines (COE-s), such as Docker Swarm [3] or Kubernetes [4]. A COE is in charge of distributing containers over multiple devices, allowing the user to treat them as a single resource cluster. In order to do so, COE-s need to determine from the user input which containers need to be executed, decide the best target location for each of them, deploy that container to the selected node, execute it, and monitor its execution to re-deploy it in case of failure [5].

Although the Cloud computing has been a game changing paradigm, there are applications that impose requirements which cannot be fulfilled by this paradigm [6]. Applications whose QoS (Quality of Service) requirements (e.g., time related constraints, security and privacy concerns, and/or energy efficiency) have a major importance are not well fitted in the Cloud paradigm. The datacenters may not be close enough to the application physical environment to provide a timely response. Data security and privacy is also a major concern in this domain, as part of the infrastructure may not be self-hosted [7,8]. Many COE-s scheduling algorithms have been designed for Cloud environments, and, therefore, their main objective when deciding where to place each container is to fit as many applications as possible, increasing the provider’s efficiency [9].

In order to provide a better fit for these applications, the Edge and Fog computing paradigms have been developed. Although these are two different paradigms, in the context of this work the distinction is irrelevant, and thus, the term Edge computing will be used (except for the related work section, where the term used by the article authors will be used). These paradigms bring Cloud computing technologies closer to the physical entities [10], where data are collected and actions are performed. Being closer to the physical world, Edge devices can offer timely responses to applications that otherwise would not be feasible through Cloud computing. On the contrary, they are not as powerful as their cloud counterparts and sometimes they must work under worse conditions in terms of availability or network reliability. As the Edge paradigm incorporates smaller nodes, edge systems can be owned by the company instead of by a third-party public Cloud provider.

Combining Edge, Fog, and Cloud computing will provide a different set of characteristics for target applications, which are required to interact with the physical world and provide a timely response, while still required to store and process high amounts of data. Therefore, the Cloud–Edge continuum offers a strong infrastructure to execute those applications. However, such a combination also introduces new challenges that need to be resolved [6,11,12,13]. First, scalability [6,11,12] and dynamicity [11,12] are considered relevant requirements to be met by these architectures. The number of nodes and concurrent applications can be very high. This issue is usually tackled by partitioning the infrastructure in smaller sub-clusters [11,12,14]. Additionally, the system faces scenarios where some of these nodes may be temporarily unavailable or new applications can be deployed. Therefore, the architecture needs to be able to adapt itself to this changing environment. Second, application QoS awareness [6,12,13] arises as an important goal for these systems, and, thus, need to be considered as part of the scheduler inputs. The QoS of an application is affected by the relative position of the related components. These relations are very important when considering applications that must met specific constraints that affect the whole application. However, existing COE-s do not consider interrelations between the containers as an input for the scheduler [9]. In order to optimize applications’ QoS, constraints that involve several containers instead of one need to be considered. Therefore, they will be denoted as inter-component constraints or requirements, in opposition to the intra-component constraints that are being considered in existing COE-s.

To address scalability, dynamicity and application QoS awareness, this paper presents ACOA (Application-Centric Orchestration Architecture), a distributed scheduling architecture targeted at the Cloud–Edge continuum. The main contributions of ACOA compared to current COE-s are:

- Generic infrastructure and workload models in order to represent the Cloud–Edge continuum devices and Edge-oriented applications, respectively;
- Distributed multi-scheduler approach to handle scalability without partitioning the infrastructure;
- Per application customization of scheduling algorithms;
- Inter-component constraint awareness at the scheduling decision making.

An implementation of ACOA has been developed on top of Kubernetes. A use-case from the railway sector is used to assess the viability of this architecture, proving that it successfully enables the use of application QoS aware scheduling algorithms in the Cloud–Edge continuum. The shift towards a distributed scheduling approach effectively tackles the scalability and dynamicity issues raised in this domain.

The layout for the remaining sections is as follows. Section 2 analyzes previous research on the field. Section 3 describes the architecture design and formally defines the infrastructure and workload models, while the architecture implementation is described in Section 4. The validation experiments carried out and their results are explained in Section 5 and some conclusions are drawn in Section 6.

## 2. Related Work

To the extent of the authors’ knowledge, modeling, monitoring, and making scheduling decisions based on applications’ QoS in Edge computing architectures is still a challenging open research topic.

From the research gaps identified throughout the previous section, several functional (FR) and non-functional (NFR) requirements for an edge oriented orchestration architecture can be extracted.

In order to take into account application QoS at scheduling time, inter-node metrics, such as the latency or the reliability of message transmission, need to be characterized (FR1). The workload definition needs to be able to represent constraints that affect a single or multiple components of each application (intra-component vs. inter-component) (FR2). Based on this infrastructure information and workload-requirements definition, the scheduling algorithm will consider both intra-component (FR3), and inter-component (FR4) requirements to determine the application’s deployment.

As inter-component relations need to be considered, application scheduling can no longer be separate scheduling decisions, one for each component. This increases the scheduling complexity and turns scalability into an important challenge for Cloud–Edge systems (NFR1). Additionally, the set of available nodes at any given time is not as stable as in a Cloud-only environment. Some of these Edge nodes may be connected through wireless technologies or not connected to the electric grid which might result in temporal lack of network connectivity or downtime in the energy supply. These variable conditions raise an important challenge: the system needs to be dynamic to adapt its deployment to these infrastructure availability changes (NFR2).

In summary, the main functional and non-functional requirements that need to be fulfilled by a Cloud–Edge oriented architecture are:

- FR1: characterize inter-node metrics;
- FR2: characterize inter-component requirements;
- FR3: schedule based on intra-component requirements;
- FR4: schedule based on inter-component requirements;
- NFR1: support system scalability;
- NFR2: enable infrastructure dynamicity.

Several research efforts have been discussed by other authors.

In [6], the authors provide a comparative analysis of four orchestration architectures and conclude that they do not solve some of the open challenges introduced by the Fog.

The work in [11] proposes an architecture that tackles the scalability issue by partitioning the infrastructure in several clusters dynamically. Despite considering QoS awareness one of the key research topics remaining, it offers no information on how the proposed architecture handles it.

In [12], an orchestration architecture is defined where fog nodes are grouped in domains supervised by a controller that is placed in a higher layer. The network state between a fog node and its controller is measured, but modeling the network state between the rest of the nodes is not addressed. It also helps with the scalability as the cluster is partitioned in several domains or groups.

In [14], an orchestration architecture for fog computing applications is developed. The infrastructure is partitioned into a single cloud and several fog regions. Latency and bandwidth measurements between each regions are measured, but monitoring the state of the network between all nodes is not addressed. It uses a throughput-aware algorithm to split applications in a Cloud and a Fog chunk that are later deployed based on their corresponding regions.

Another Cloud-oriented architecture is developed in [15], where scheduling is based on labels that define node characteristics and application requirements. The defined labels are static (defined by the user when a node joins the system) and qualitative. How to dynamically update these labels is an open question. One of the labels splits the infrastructure in three different layers (Cloud, Fog, and Edge), tackling scalability by partitioning the infrastructure. They verify their architecture with a video streaming application that uses this layer label to configure its deployment.

In [16], current virtualization and orchestration frameworks are evaluated, focusing on scalability and orchestration capabilities. A centralized orchestration architecture is proposed. Although node monitoring is considered, network monitoring is not part of the scope of this paper.

The work in [17] provides a full workload and infrastructure model. Edge and Cloud nodes are modeled differently, splitting the infrastructure in two different layers. An algorithm is also provided that is able to filter the valid deployments according to inter-component restrictions. No mechanism is provided to rank the valid deployments, this decision is voluntarily left to expert opinion.

In [18], an architecture for service orchestration in Fog-enabled infrastructures is suggested. Despite including some monitoring components, monitoring inter-component metrics is not addressed, being unable to make orchestration decisions based on application’s QoS requirements.

In [19], an architecture for micro-service deployment is designed. Micro-services that communicate with each other are collocated in order to reduce the communication overhead between them. However, the network state between the nodes is not being considered. It dynamically detects congested nodes and performs components migrations, but the scalability of the system is not being addressed.

The architectures above support some of the requirements extracted previously, but none of them fulfills all of them. A summary of the requirement fulfillment is depicted in Table 1.

Additionally, several works have focused on network-aware orchestration algorithms. These algorithms demonstrate how the use of the network state during scheduling decisions can be used to optimize applications’ QoS.

The work in [20] presents a network-aware orchestration algorithm designed for Cloud computing environments. The authors optimize several parameters, such as network bandwidth or latency. In [21], a cost-aware and delay-constrained algorithm is provided. The algorithm and simulation are designed for multi-datacenter networks related to Cloud computing. Ref. [22] describes a set of orchestration algorithms for communication provider’s networks. One of these algorithms minimizes network latency. This algorithm is evaluated in a test-bed with five emulated datacenters. Finally, a Fog oriented heuristic algorithm for smart production lines is developed in [23]. It determines tasks placement to minimize delay and energy consumption.

In order to enable the use of these algorithms in the Cloud–Edge continuum, the scheduler needs to be provided with network state metrics. Previous work of the authors [24] presented SWIM-NSM (SWIM Network State Monitoring) protocol to characterize network-related metrics (FR1). SWIM-NSM is based on the SWIM protocol (Scalable Weakly-consistent Infection-style process group Membership protocol) [25] that allows a scalable way (NFR1) of maintaining an up-to-date list of the nodes in a cluster. The presented protocol adds mechanisms to capture network related metrics for every pair of nodes, while maintaining the same focus as the original protocol on being lightweight and scalable.

Another previous work of the authors [26] also investigated the distribution of the scheduling logic among several schedulers instead of a single centralized one. Multiple parallel schedulers increase the scheduling capacity of the architecture which tackles the scalability issue (NFR1). The trade off of distributed scheduling is that some scheduling decisions made in parallel may conflict with each other, resulting in a scheduling collision that requires the algorithm to be executed again.

## 3. Distributed Scheduling Architecture for the Cloud–Edge Continuum

As identified in Section 2, none of the current architectures fulfill all the requirements that have been considered for ACOA. Scalability (NFR1) is broadly considered one of the challenges of orchestration in the Edge, as recurrently mentioned in the related work. The most common approach to tackle the scalability issue is by partitioning the infrastructure into separate smaller groups of nodes [6,11,12], instead of a global one. Application components will be placed in one of these partitions, either by user interaction or by an automatic pre-scheduling process, and then scheduled in one of the nodes of that partition.

When taking into account the inter-component requirements (FR1, FR2, and FR4) of an application, a scheduler cannot make decisions on components alone; it has to do it in an application basis, i.e., applications are the minimum scheduling unit, not components. When following the divide-and-conquer approach of partitioning, applications would be restricted to a single partition, and thus the deployments will be sub-optimal. For this reason, a single-partition approach has been used, and the scalability issue has been considered in the whole architecture, from infrastructure monitoring to scheduling decision making.

Next, the main system components of ACOA and their distribution in the different planes and layers are described. The workload and infrastructure models are also presented.

### 3.1. Architecture Components

**Figure 1:**
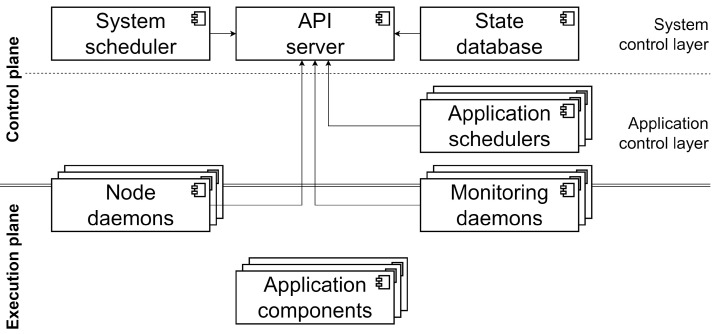
Architecture design.

The architecture is divided into two different planes: the control plane and the execution plane, as depicted in Figure 1. The control plane will perform the architecture management related tasks, and is divided in the system control layer and application control layer. The execution plane is where the application components will be deployed. When a node joins the system, it must specify which planes and layers it is part of. Having multiple nodes in each control plane layer increases the scalability (NFR1) and dynamicity (NFR2) of the system.

The description of each of the system components can be found below. They are deployed as containers, and can be divided in three categories:

- System control components: deployed in every node that joins the homonymous layer. There are three system components belonging to this category: the API server, the state database and the system scheduler;
- Application control components: deployed in the corresponding layer as required by the system’s workload. Application schedulers are the only system component that belongs to this category;
- System daemons: deployed in every node that joins the system, independently of which layers it is part of. There are two types of system components that belong to this category: the node daemon and the monitoring daemon.

The **API server** is the system control component that allows the remaining of the system components to interact among them through a REST API. As the rest of system control components, the API server is part of the system control layer. When several nodes are part of this layer, multiple API server instances will be present, which improves the performance and resilience of this system component (NFR1 and NFR2). Additionally, user interaction with the system, e.g., deploying an application, will also be performed through the API server by providing YAML definitions of the desired outcome.

The **state database** is a system control component, therefore, part of the system control layer. The database will contain the information about the state of the system. It comprises both the current state of the nodes, network, and deployed components, as well as the applications defined by the users. The end goal of the whole system will be to take the necessary actions in order to reconcile the current state with the desired state obtained from the user input. By using a distributed database, multiple instances of this system component will improve the performance and resilience (NFR1 and NFR2).

The **system scheduler** is the remaining system control component, part of the layer with the same name. The system scheduler is in charge of deploying new schedulers in the application control layer when new applications are deployed (NFR2), as described below. When several nodes are part of this layer, one of the system scheduler instances will be active while the rest will be idle for resilience purposes, but the performance remains the same.

**Application schedulers** are application control components, so they are deployed in the application control layer. These application schedulers will make use of the state information available in the state database in order to select the nodes in which the application components will be deployed to maximize its QoS. Each application will have its dedicated scheduler. This means that applications will have control over which scheduling algorithm should be used.

**Node daemons** are system daemons that are in charge of executing and monitoring the containers of their corresponding node. They are deployed in every node at startup, as they are used to deploy both system and application components.

**Monitoring daemons** are deployed in every node in order to monitor their state and their surrounding network. The constantly monitored state of the infrastructure is used to update the state database. Node state is defined by dynamic properties such as the amount of available memory or the CPU load of a node. Network state is monitored with the SWIM-NSM protocol presented in [24]. This protocol allows collecting latency, jitter, and success or fail ratio measurements for every pair of nodes in the system (FR1). All these monitored data will later be used by the application schedulers to make their decisions (FR3 and FR4).

### 3.2. System Modeling

In order to store inter-node metrics (FR1) and define inter-component relationships (FR2), the infrastructure (nodes and network links among them) and workload (applications and their requirements) will be formally modeled in the following sub-section. These models will be updated as the system evolves and are used by ACOA to make deployment decisions.

#### 3.2.1. Infrastructure Model

**Figure 2:**
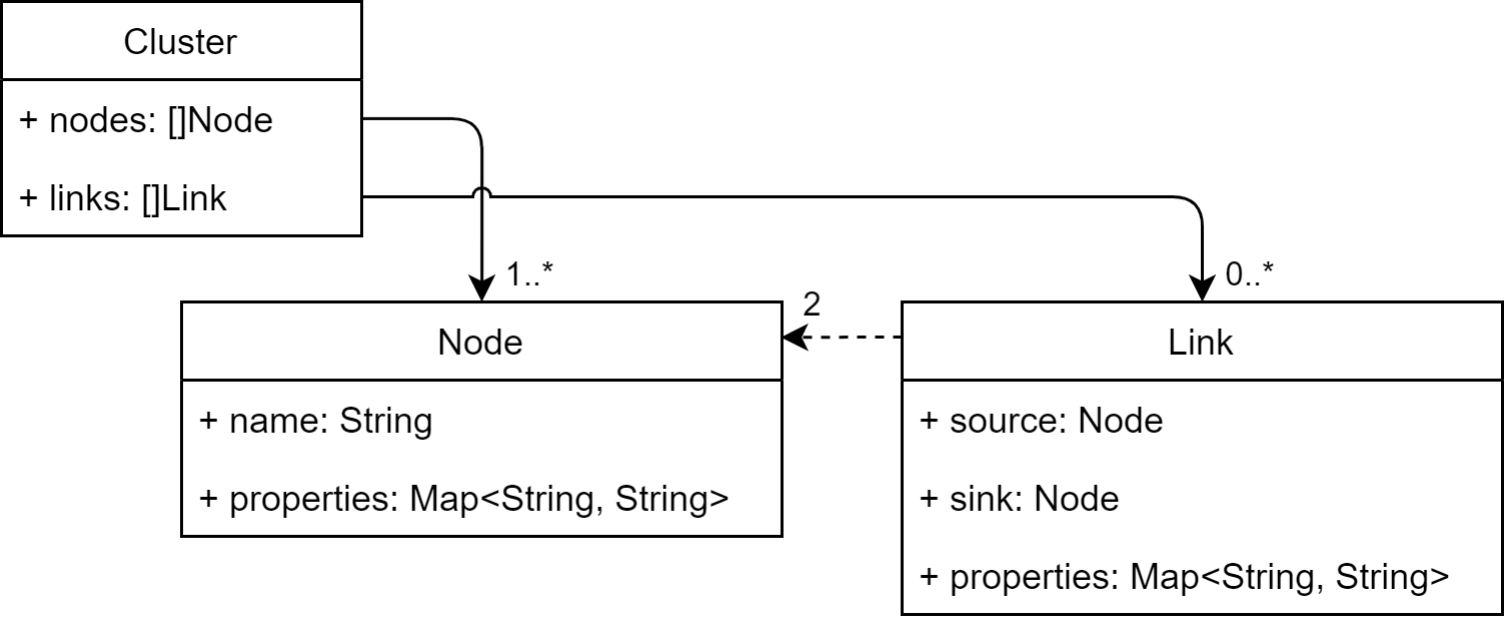
Infrastructure model.

The infrastructure model is depicted in Figure 2. The aim of this model is to represent nodes throughout the Cloud–Edge continuum by providing a single generic model instead of several more specialized ones for each layer. This avoids the need of partitioning the infrastructure by layers to achieve the desired single-partition infrastructure.

The whole set of **nodes** that are part of the partition are grouped in one **cluster**. Each node is identified by a unique name within the cluster, and is defined by a set of static and dynamic properties. On the one hand, static properties represent characteristics that do not change, or at least do not change often, e.g., the operating system, whether a disk is encrypted, or the datacenter which this node belongs to. On the other hand, dynamic properties represent characteristics that experiment variations over time, such as the available memory or the CPU load.

The network is modeled by a set of network **links** between a source node and a sink node. The need of a **link** model is raised by the inclusion of inter-node metrics (FR1). The direction of the link is important to represent the network asymmetry, thus the total number of **links** is two times the square of the number of nodes. Each network **link** has a set of properties, e.g., latency, jitter, or the transmission success rate.

Dynamic properties, both for **nodes** and network **links**, will be updated continuously by the aforementioned monitoring daemons to ensure that their values are up to date. All these properties will be available as inputs to the application scheduler in order to make the optimal deployment decision. These properties can be extended if the scheduler requires additional inputs.

#### 3.2.2. Workload Model

**Figure 3:**
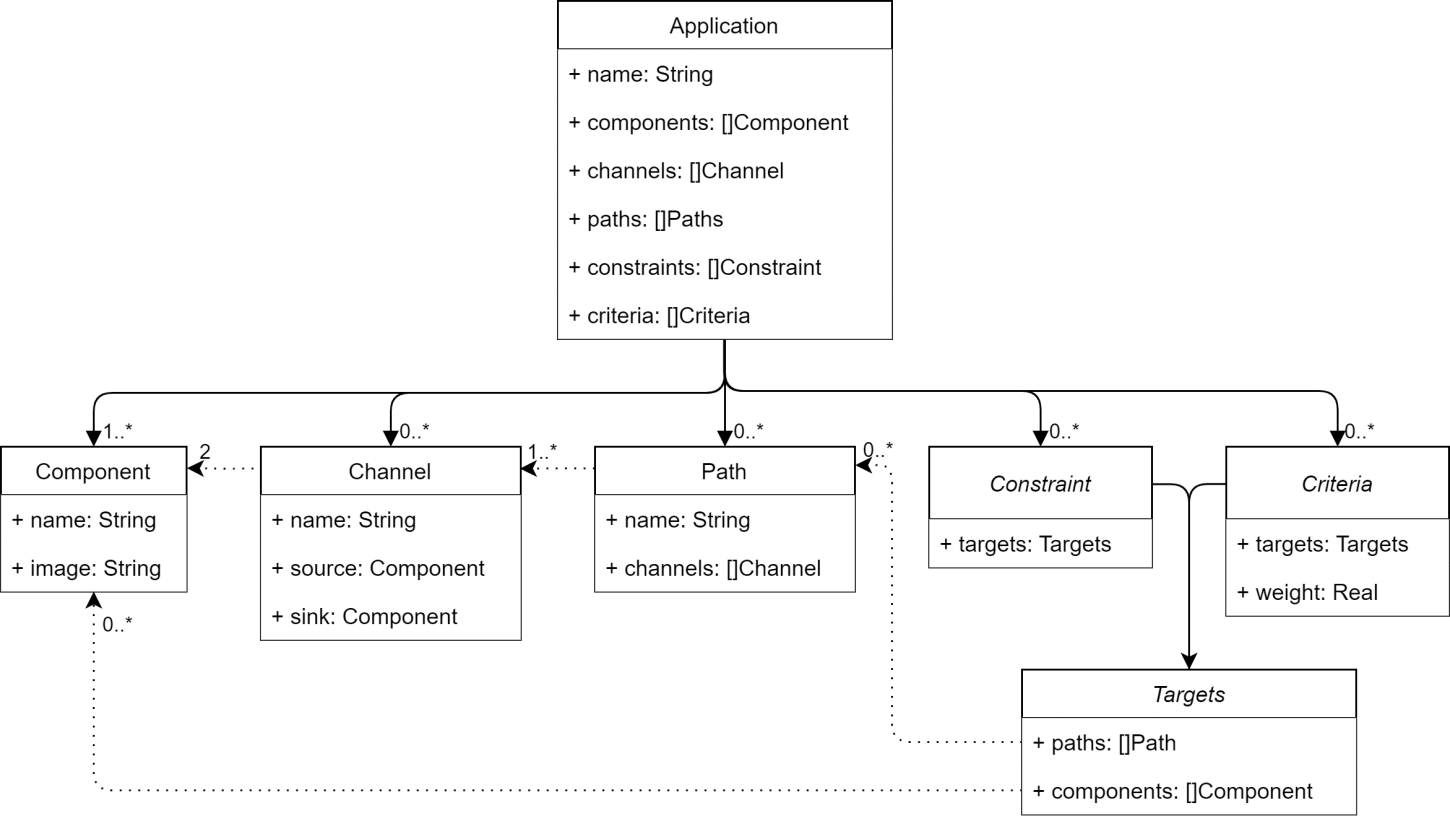
Workload model.

The workload model has been designed to be generic enough to include all kind of applications that could fit in the Cloud–Edge continuum, while still providing all the information required by the architecture to schedule based on the application specific QoS. Figure 3 shows the workload model that represents **applications** that will be executed by the system.

Multiple **applications** will be run by the system, so they need a unique name that will act as an identifier. They are composed of **components** which represent the executable pieces that will perform the application’s tasks. **Components** also require a unique name per application, and, as they are realized by containers, they require further information to identify the container image that implements each task, provided as a path. **Channels** define the messages transmitted from a source to a sink **component**, and also require a unique name per application. Defining a source and a sink component allows defining the direction of the messages, as the network cannot be assumed to be symmetric. Therefore, **applications** can be represented as DGs (Directed Graphs), where **components** are the graph’s vertices and **channels** are the edges.

Most of the current orchestration engines allow setting constraints on which nodes a certain component can be deployed. They will later use a cluster-wide policy (e.g., spreading the workload among all the nodes) and resort to randomness in case of a tie. This work considers two kinds of policies to customize the deployment of an application. **Constraints**, the first policy type, filter the potential nodes where each component can be deployed. Several types of constraints have been defined: requiring a certain hardware (sensor/actuator) or software (database) component or a certain amount of free disk space. Optimization **criteria**, the second policy type, allow configuring, in an application basis, how the exact node from the set of potential targets is chosen. Multiple optimization criteria can be provided with different weights to achieve the desired deployment behavior for each application separately, thus allowing its QoS to be optimized. Several optimization criteria types have been defined: prioritizing the nodes with the less number of containers, the highest percentage of free memory, minimizing the end-to-end (e2e) response time for a certain path, or maximizing the message transmission success rate. As the system evolves, new policies, both constraints and optimization criteria, will be defined to represent other types of applications’ QoS.

Both **constraints** and optimization **criteria** need to define their **targets**. They can be applied in a per-component basis or to subsets of the application (e.g., minimizing the e2e response time of an application critical path needs to define the critical path). These subsets are defined via **paths**, which are referenced by a unique name within an application. They are characterized by the **channels** that join a set of **components**, which are implicitly included in the **path**. As **paths** are subsets of the application, they also can be represented as DG. Furthermore, some policies may impose additional requirements to these graphs, such as forming a DAG (Directed Acyclic Graph), e.g., the response time optimization criteria mentioned above requires the path to be acyclic, as cycles will create loops that will result in infinite recursion when computing e2e measurements.

This model allows the users to define their applications’ components, relations, and QoS (FR2). This application definition will be used by the application scheduler in conjunction with the monitoring data from the infrastructure model to determine the optimal deployment for each of the components.

### 3.3. Distributed Scheduling

As described above, a distributed scheduling approach is used in ACOA. Each application will have its dedicated scheduler customized to its needs by the policies described in the workload model. This distributed approach is an improved version of a previous work that authors presented in [26]. It has been designed focusing the scalability (NFR1) of the scheduling process.

Applications are deployed in two steps. First, an application scheduler tailored to that application is deployed. Next, the application scheduler is used to deploy the application components in the nodes that better fit their QoS requirements. The processes followed to deploy new application schedulers and components when new applications are instantiated are described below. The scheduling algorithm used to minimize e2e response time and maximize e2e reliability is also described.

#### 3.3.1. Application Scheduler Deployment Process

**Figure 4:**
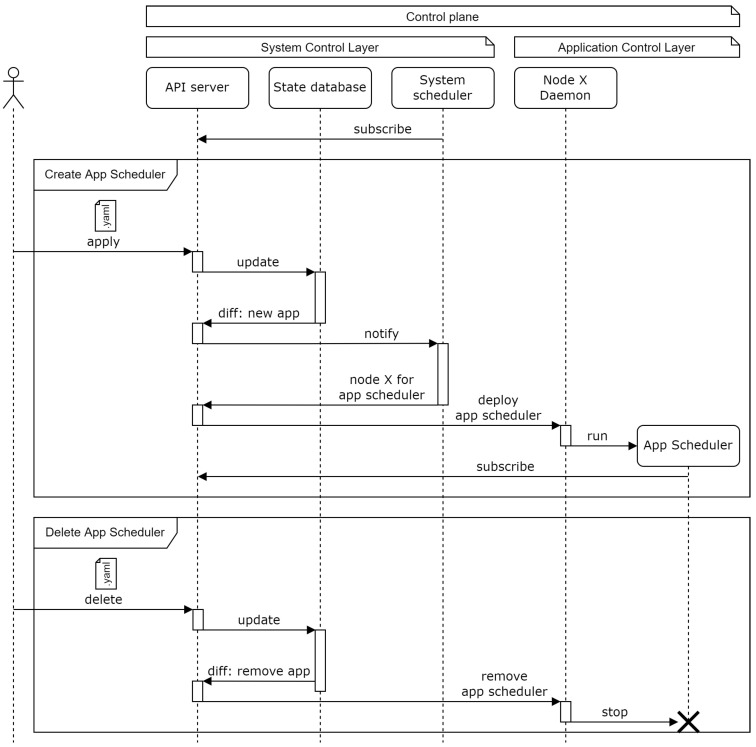
UML sequence diagram for applications’ schedulers deployment.

These distributed schedulers are deployed dynamically as new applications are instantiated by the user. Figure 4 shows the sequence diagram of this process.

When a user defines a new application through the API server, the state database is updated and the changes are reported back to the API server. The system scheduler is notified and chooses a target node (among the application control layer nodes) where the new application scheduler should be deployed. The order is sent to the selected node which runs the corresponding container.

When an application becomes removed from the system, the reverse process is executed. The state database is updated and the application scheduler that needs to be deleted is reported back to the API server. This application scheduler is removed by the corresponding node daemon without the interaction of the system scheduler.

#### 3.3.2. Application Component Deployment Process

**Figure 5:**
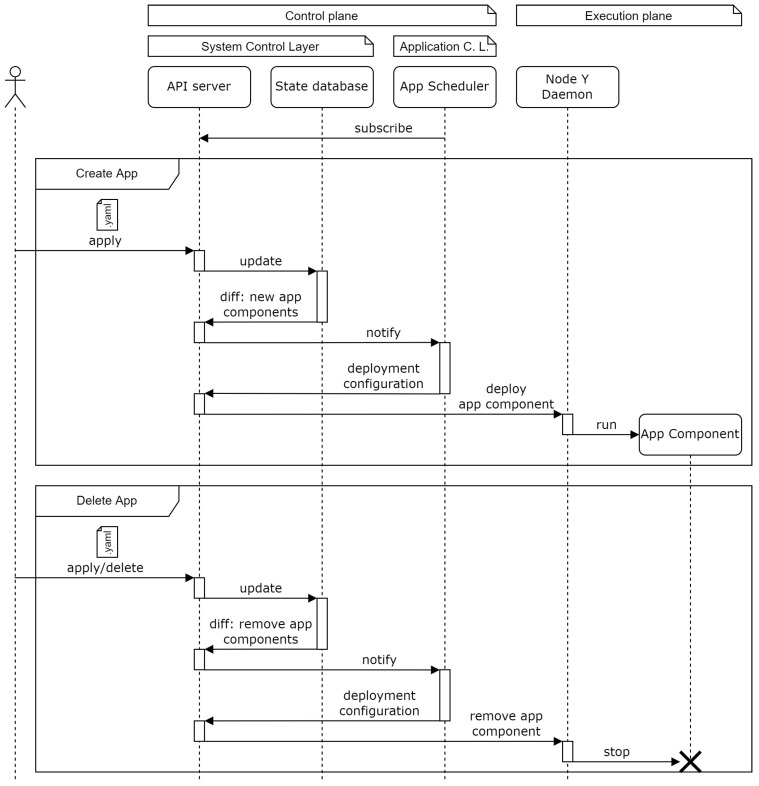
UML sequence diagram for applications’ components deployment.

Once the application scheduler is deployed, the application components can be deployed according to the sequence diagram in Figure 5.

Either when the initial application is applied, or when a further application update includes new components, the state database is updated and the differences from the previous state are reported back to the API server. The application scheduler issues a new application deployment configuration for each of the application components based on the state database data. The corresponding orders are sent to the selected node daemons which will run the required containers.

The same process is followed when an application component needs to be deleted, either because of an application delete operation or due to an application apply operation lacking one of the previously existing components.

#### 3.3.3. Application Scheduler Algorithm

An application scheduler has been designed that can minimize the e2e response time and maximize the e2e reliability. The optimization criteria defined in the workload model are used to score the possible deployments. Each of these criteria (*j*) will be evaluated to obtain a grade between 0 and 1 for each possible deployment (*i*) which will be denoted as $g_{i,j}$.

In order to minimize the e2e response time, the accumulated latency for the corresponding path is calculated by adding the individual steps. In order to obtain a grade between 0 and 1, the minimum accumulated latency for all possible deployments is calculated. The minimum accumulated latency is divided by the accumulated latency of each possible deployment to obtain the grade corresponding to that deployment as shown in Equation (1). $l_{j}^{min}$ denotes the minimum accumulated latency and $l_{i,j}$ is the accumulated latency for the deployment *i*.

(1) $$g_{i,j}=\frac{l_{j}^{min}}{l_{i,j}}$$

Maximizing the e2e reliability makes use of the accumulated success rate. It is computed by multiplying the individual values of each of the steps. Success rates are already between 0 and 1, so their product can be used directly as the grade for each deployment as seen in Equation (2). $s_{i,j}$ denotes the accumulated success rate for deployment *i*.

(2) $$g_{i,j}=s_{i,j}$$

Once all optimization criteria have been used to compute their grades, a final grade is calculated using Equation (3). $G_{i}$ denotes the final grade for the deployment *i* and $w_{j}$ is the weight of the optimization criteria *j*. The deployment with the highest grade is chosen.

(3) $$G_{i}=\sum^{\forall j}w_{j}g_{i,j}$$

ACOA can be extended with additional scheduling algorithms implemented in containers. The complexity of these algorithms can be very different, depending on the applications needs. They can use complex scheduling algorithms as the ones described in [20,21,22,23].

## 4. Implementation

The proposed architecture has been implemented atop Kubernetes. Kubernetes architecture does not fulfill all the requirements established for the Cloud–Edge continuum applications in the previous section. In order to fulfill these requirements, the vanilla Kubernetes architecture has been extended with new node types and system components. In this section, the Kubernetes architecture is described first, and then, the modifications in order to implement the proposed architecture are explained.

### 4.1. Brief Overview of Kubernetes Architecture

Kubernetes (also called k8s) [4] is a mature orchestration platform and one of the most used COE-s in Cloud computing environments. For the sake of understanding the implementation of ACOA, a brief Kubernetes overview is included in this section.

Kubernetes provides an abstraction layer over containers called pods. A pod allows defining a set of one or more containers that need to be deployed together with their context, resource, and network specification. Additionally, Kubernetes also provides extra abstractions that implement functionality, such as replicating a pod several times (replica set) or providing rolling updates (deployment).

In Kubernetes, nodes are spread into the control and execution planes, dividing them in two different types: control plane nodes and execution nodes. Optionally, control plane nodes can also be part of the execution plane. Multiple control plane node setups are possible, and this kind of configuration are called “high availability setups”, as they provide redundancy for system-control components.

Five main system components are used to implement the Kubernetes architecture, four of them placed in the control plane and the last one in the execution plane where the workload will be run. The four control plane components are the API server, the ETCD database, the controllers, and the scheduler. The fifth component is the kubelet.

Communication with the k8s system is completed through the API server. It also coordinates the rest of the system components. The ETCD database is a distributed NOSQL database that stores all the information about the infrastructure and the workload. These two first system components increase their performance when multiple nodes are deployed in the control plane of a cluster, providing higher processing capabilities for API calls, and increased storage space.

The controllers are in charge of implementing the Kubernetes model, translating the abstractions (replica sets, deployments, ...) into pods. These pods are then processed by the scheduler and assigned to one of the nodes in the execution plane. These two last components do not benefit from increased performance when a high availability setup is configured. Instead, only one of the replicas of each component is active while the rest wait idle to take the place of the active one in the event of some error. This redundancy increases availability and fault tolerance, but not performance.

The fifth system component is the kubelet, which is placed in every node and is part of the execution plane. It handles the communication with the Kubernetes system through the API server for both reporting the state of the node and executing the pods deployed by the scheduler.

### 4.2. Architecture Overhaul

ACOA and Kubernetes are both divided into two different planes: the control and the execution planes. These planes are represented by the two different node types in Kubernetes. However, ACOA further splits the control plane into two different layers, requiring a third node type. This new node type will represent the application control layer while the previous control plane nodes will represent the system control layer.

**Figure 6:**
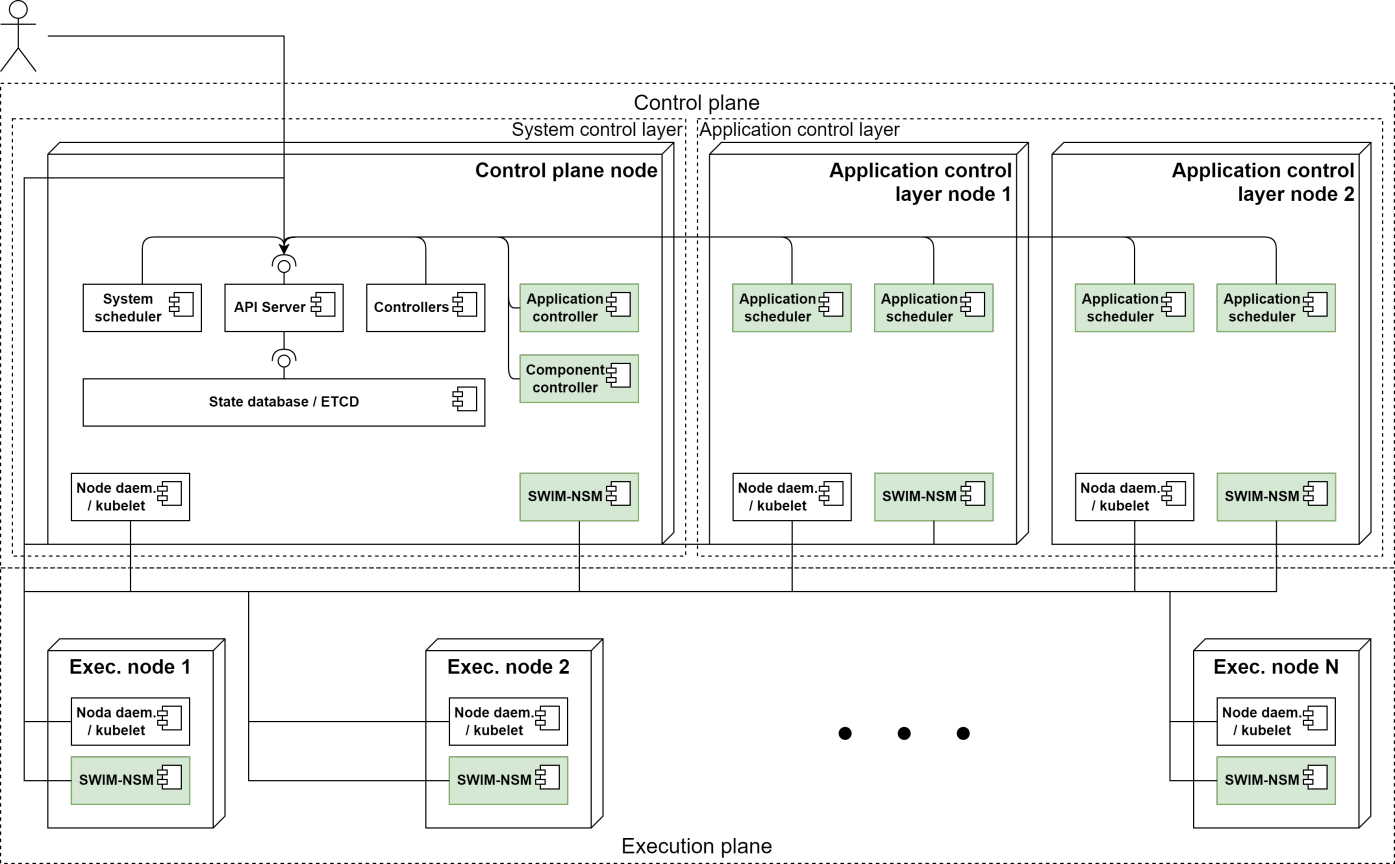
UML component diagram of ACOA architecture over K8s.

Some of Kubernetes components directly relate with the ACOA system components described in Section 3. The k8s API server and its controllers perform ACOA’s API sever tasks, but additional controllers need to be implemented to represent the new abstractions introduced by the proposed workload model. The ETCD database is used as the state database and k8s’s scheduler is used as the system scheduler in ACOA. New scheduler components need to be deployed to implement the application schedulers. The kubelet performs ACOA’s node daemon tasks. Additionally, it also monitors some node metrics, but additional monitoring daemons need to be deployed to monitor the network state. The resulting architecture diagram is depicted in Figure 6, where the new components have been highlighted and are described below.

#### 4.2.1. Application Control Layer Nodes

In order to implement the described architecture and distribute the scheduling logic, additional nodes will be part of the control plane. Previously existing Kubernetes control plane nodes realize ACOA’s system control layer, while these additional nodes will realize the application control layer, running the distributed application schedulers described in Section 3.

Similarly to the previously existing Kubernetes control plane nodes, they can be part of the execution plane. Making control plane nodes also be part of the execution plane is useful for infrastructure setups with a smaller number of nodes where dedicating nodes to the control plane may be too much overhead.

#### 4.2.2. Application and Component Controllers

Kubernetes neither provides an application model nor takes into consideration inter-component relationships (FR2). Consequently, it needs to be extended with the models presented in Section 3.

Similarly to how k8s controllers implement the functionality of the abstractions (replica set, deployments, ...), two new controllers are deployed to implement the aforementioned workload model. The application and component controllers translate the application definitions provided by the user into pods, that will be scheduled by the application schedulers below.

#### 4.2.3. Application Schedulers

In order to base the scheduling decisions, not only on component requirements (FR3), but also on inter-component ones (FR4), new application schedulers will be deployed, as illustrated in Figure 6. The deployment of a new application scheduler in the application control layer nodes when a new application becomes instantiated is performed as described in Section 3.

Each of these application schedulers will be in charge of choosing the best deployment for an application defined by the user. In order to do so, they will take into account the different application QoS defined as constraints and optimization criteria in the workload model and the monitoring information from the infrastructure model.

#### 4.2.4. SWIM-NSM Daemon

Kubernetes’ kubelet daemon already monitors the node state. In order to monitor the network state, the SWIM-NSM protocol presented in [24] is used. This protocol allows collecting network-related metrics for every pair of nodes in the system.

A daemon with an implementation of this protocol is deployed in every node of the system, as seen in Figure 6. It updates the system inter-node metrics (FR1) with latency, jitter, and success or fail rate measurements between every pair of nodes, updating the dynamic properties of link objects modeled in Section 3.

## 5. Evaluation

The railway sector is considered a vertical domain where resources throughout the whole Cloud–Edge continuum are required. Aside from Cloud datacenters, where the heaviest part of processing and storage can be completed, computer and data nodes can also be found in trains and stations (Edge computing).

Multiple applications are being executed in railway scenarios: smoke detection, verifying that doors are closed before departure and speed profiling, among others. Speed profiling assigns each train the speed profile that it needs to follow in order to fulfill its planned schedule based on the position of other trains and the situation of nearby stations.

**Figure 7:**
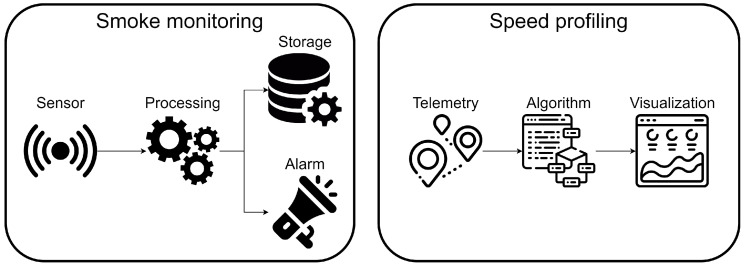
Railway applications.

In order to validate the proposed architecture, the smoke monitoring and speed profiling applications depicted in Figure 7 will be deployed. These applications showcase some of the capabilities of ACOA: the introduction of the application concept to the scheduling algorithm, the customization of the algorithm in a per-application basis and the usage of inter-component metrics like the e2e response time and reliability mentioned in the previous section.

### 5.1. Infrastructure

In order to deploy these applications, 20 nodes have been provisioned that act as ACOA nodes in a single cluster. They are running k3s, a certified lightweight implementation of Kubernetes “built for IoT & Edge computing” [27]. All the nodes are fully connected in a mesh topology. They represent five different datacenters: two cloud datacenters of six nodes each, two trains with three nodes each, and a station with two nodes. The distribution is shown in Table 2.

Nodes corresponding to Cloud datacenters (1–12) have the needed resources for more demanding components such as historic database storage or speed profiling algorithm execution. The first node in each train (13 and 16) has access to the security data, including the measurements from the smoke sensors. The second node (14 and 17) represents the driver’s dashboard where relevant information is displayed. The last node in each train (15 and 18) is part of the telemetry system, including GPS coordinates and speed measurements. These aspects have been modeled as the corresponding node static properties described in Section 3.

The 20 provisioned nodes used for this validation are in the same physical location despite representing 5 different datacenters. The measurements of the SWIM-NSM daemon cannot be used for this assessment. A simulated network monitoring system has been used instead of the real SWIM-NSM daemon. The simulated values for latencies and success rates are randomly generated taking into account if they are measuring the network with itself, or if they both belong to the same or different datacenters. The lower and upper bounds have been selected based on previous measurements for these kinds of connections and are summarized in Table 3. These values are being updated in a periodic fashion and stored in the dynamic properties of the links described in Section 3.

All this information is available to ACOA as part of the infrastructure model defined in Section 3.

### 5.2. Workload

Two different applications are deployed simultaneously. The smoke detection application corresponding to the first train and the speed profiling application that involves both trains.

#### 5.2.1. Smoke Monitoring Application

**Figure 8:**
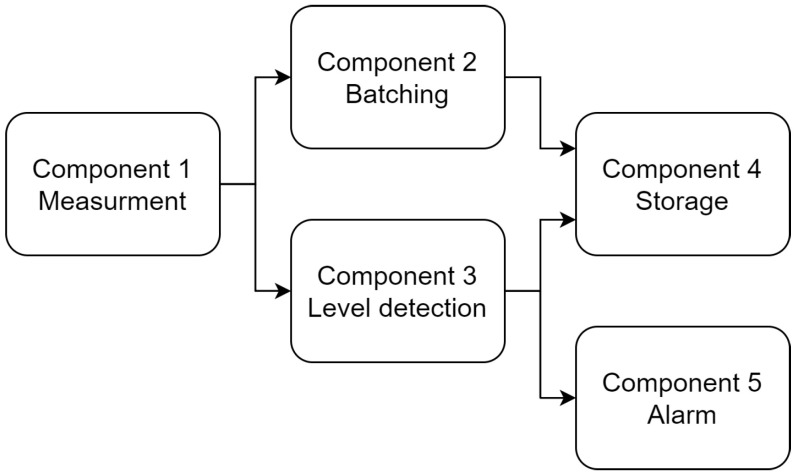
Smoke monitoring application.

A sensor measures the smoke concentration in a wagon. The measurements are stored in a database after being processed, and are also used to raise an alarm in the driver’s dashboard if they go over a certain threshold. Despite being a simple application, it includes preprocessing, historic data storage in the Cloud and short deadlines for alarm triggering, illustrating some of the most common requirements of applications targeted towards the Cloud–Edge continuum. The application has been implemented with five components as depicted in Figure 8:

- Component 1 (Measurement) reads the measurements of the smoke concentration from the sensor for the next two components;
- Component 2 (Batching) groups multiple measurements and computes deltas to reduce the memory footprint required to store the data;
- Component 3 (Level detection) triggers events when the values go above or below certain thresholds;
- Component 4 (Storage) stores the data and the events into a database;
- Component 5 (Alarm) fires an alarm to inform that the configured threshold was surpassed.

Some of these components impose some constraints that limit the nodes where they can be deployed. Component 1 requires the node to have accessed to the smoke sensor, Component 5 requires the node to have access to the driver’s dashboard, and Component 4 requires the node to be able to access a database. These constraints are depicted in Table 4.

Aside from these constraints, additional policies have been defined to improve the application’s QoS as defined in Section 3. In order to define these policies, three different paths have been defined:

- Data storage: comprises components 1, 2, and 4.
- Event storage: comprises components 1, 3, and 4.
- Alarm: comprises components 1, 3, and 5.

Triggering the alarm in a timely and reliable fashion is the most critical path, and, thus, policies for minimizing the latency and maximizing the reliability will be configured. Additionally, both storage paths do not have any time constraint, so no policy will be placed in this case. The data storage path will have a policy maximizing its reliability, but with a weight four times smaller than the two previous ones as it is less important. As the event information can be reconstructed from the data, the event storage path will not have any reliability policy either. These optimization criteria have been depicted in Table 5.

#### 5.2.2. Speed Profiling Application

**Figure 9:**
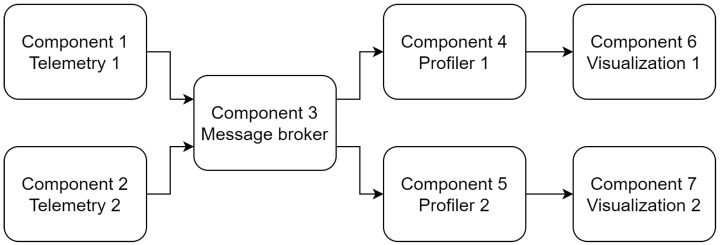
Speed profiling application.

Speed profiling application’s goal is to determine which is the optimal speed for a train based on all the information available. This includes, among other data, the expected arrival time and the position and speed of other surrounding trains. Computing these speed profiles can prove to be resource demanding for nodes in the train and is offloaded to Cloud nodes. The application has been implemented with seven components as depicted in Figure 9:

- Component 1 and 2 (Telemetry) read the GPS coordinates and speed measurements of each train that are required by the speed profiling algorithms;
- Component 3 (Message broker) enables the transmission of the gathered data to all of the profiler algorithms through a publisher–subscriber pattern;
- Component 4 and 5 (Profiler) compute the optimal speed profiles that each train should follow based on all the information they have;
- Component 6 and 7 (Visualization) show the optimal speed profile that the driver of each train needs to follow.

Some of these components impose some constraints that limit the nodes where they can be deployed. Component 1 and 2 require the node to have accessed to the train’s telemetry data, Components 4 and 5 require a Cloud node with high processing power and Components 6 and 7 need to have access to the driver’s dashboard. These constraints are depicted in Table 6.

Aside from these constraints, additional policies have been defined to improve the application’s QoS as defined in Section 3. In order to define these policies, two different paths have been defined, one for each train:

- Train 1: comprises components 1, 3, 4, and 6.
- Train 2: comprises components 2, 3, 5, and 7.

The use of a message broker and the publisher–subscriber pattern already provides reliability guarantees. Being able to react quickly to unexpected changes, such as delays of surrounding trains, is key for the QoS of the speed profiling application. Each of the paths will have configured a policy minimizing the e2e response time. These optimization criteria have been depicted in Table 7.

All this workload information has been used to create the workload model as defined in Section 3. This model is provided to ACOA in the form of a YAML file. This file contains, among other details, the location of the containers that implement each of the components of the applications, the constraints depicted in Table 4 and Table 6, and the optimization criteria summed up in Table 5 and Table 7.

### 5.3. Results

In order to compare the behavior of the proposed architecture, the same applications have been deployed in the aforementioned infrastructure using both base kubernetes and ACOA. Both apply the component constraints, while only ACOA is able to apply the optimization criteria policies mentioned above.

As the scheduling decision made by the proposed architecture is based on the mentioned optimization criteria, the deployment is deterministic for a given network state. This does not apply to the base k8s scheduler, which ends up defaulting to picking a random node among those that have not been already selected for another component. This results in 4800 potential deployments for the smoke monitoring application and 2880 potential deployments for the speed profiling application.

The best and worst potential deployments for the smoke monitoring application, as graded by the aforementioned policies, have been depicted in Table 8. ACOA will select the best graded deployment. Having 4800 potential deployments, the chance that the default Kubernetes scheduler chooses the same deployment as ACOA is 0.02%. As expected, Components 1 (measurement) and 5 (alarm) are fixed to nodes 13 and 14, respectively, as they are dependant on some hardware that is only accessible by those nodes. Component 2 (batching) and Component 4 have been deployed in Cloud nodes in both cases, being the difference between them that the worst case places them in separate Cloud datacenters, while the best case places them in the same node. Component 3 (level detection) has been deployed in the Cloud too for the worst case scenario, while it is kept in the train for the best case. This means two extra hops from/to the Cloud for the worst case scenario which explains the low score for this case.

Similarly, the best and worst potential deployments for the speed profiling application have been depicted in Table 9. ACOA will select the best graded deployment. The number of potential deployments is a bit lower, 2880, resulting in a 0.03% chance of Kubernetes choosing the same scenario as ACOA. As expected, Components 1 and 2 (telemetry), and Components 6 and 7 (visualization) are fixed to nodes 15, 18, 14, and 17, respectively. The rest of the components have been deployed in Cloud nodes for both the best and the worst cases. The difference, similar to the smoke monitoring application’s data path, is that the best case scenario deploys all of them in the same Cloud datacenter, while the worst case scenario deploys the message broker in a different datacenter from the two profilers. This requires an additional hop between datacenters that is more expensive time-wise.

Despite not happening during this validation, it must be noted that the main drawback of a distributed scheduling approach is the apparition of collisions. A collision happens when two schedulers running in parallel make a deployment decision that conflicts with each other. This may result in requesting more resources that those that a node has at its disposal, requiring one of the conflicting schedulers to compute a new deployment. In this regard, the authors demonstrated in a previous work [26] that, although the number of collisions with multiple schedulers in parallel is rare, the reduction in the mean scheduling time is significant.

## 6. Conclusions

Edge computing applications rise new challenges for orchestration engines. More complex decision based on per-application policies are required to guarantee application’s QoS across Cloud–Edge continuum, as Edge nodes do not have the same stable conditions that a Cloud node has. The scalability and dynamicity of this paradigm also need to be taken into account.

This paper proposes a scalable orchestration architecture that distributes the scheduling tasks among several nodes of the system in contrast with the centralized approach usually followed by current architectures. A generic model for the infrastructure is defined that can fit very heterogeneous devices, from high-performance Cloud-oriented hardware to smart sensors and actuators in the Edge. A workload model is also proposed that allows describing applications as a set of components and their relationships, allowing the definition of constraints and optimization criteria policies that maximize the applications’ QoS.

ACOA has been implemented over Kubernetes and has been validated for two applications of the railway domain in a multi-datacenter infrastructure setup. The results of this case study show how this architecture is able to improve the QoS of the applications by intelligently selecting the nodes where each component will be run.

The scheduling algorithm uses inter-node metrics, such as latencies and message success rates. More complex and precise algorithms have been developed throughout the last decades that require these inter-node metrics for application planning. The proposed architecture enables the usage of these algorithms configured in a per-application basis. The implementation of these algorithms is an on going process.

## Figures and Tables

**Table 1:** Related work comparison.

Authors	Ref.	FR1	FR2	FR3	FR4	NFR1	NFR2
K. Velasquez et al.	[6]	✗	✗	✓	✗	Partitions	✓
Z. Wen et al.	[11]	✓	✗	✓	✗	Partitions	✓
Y. Jiang et al.	[12]	✗	✓	✓	✓	Partitions	✓
F. Faticanti et al.	[14]	✗	✓	✓	✓	Partitions	✓
C. Wöbker et al.	[15]	✗	✗	✓	✗	✗	✗
S. Hoque et al.	[16]	✗	✗	✓	✗	✗	✗
A. Brogi et al.	[17]	✗	✓	✓	✓	✗	✗
M. S. de Brito et al.	[18]	✗	✗	✓	✗	✗	✗
K. Fu et al.	[19]	✗	✗	✓	✓	✗	✓

**Table 2:** Nodes distribution among datacenters.

Datacenter	Nodes
Cloud 1	1–6
Cloud 2	7–12
Train 1	13–15
Train 2	16–18
Station	19–20

**Table 3:** Network metrics bounds.

Target	Latency	Success Rate
Itself	0.25–0.35 ms	100%
Same datacenter	0.8–1.2 ms	95–100%
Different datacenter	15–25 ms	85–95%

**Table 4:** Smoke monitoring application constraints.

Component		Hardware	Software	Possible Nodes
1	Measurement	Sensor		13
4	Storage		Database	1–12
5	Alarm	Dashboard		14

**Table 5:** Smoke monitoring application optimization criteria.

Path	Optimization Criteria	Priority	Weight
Data storage	Maximize e2e reliability	Low	0.25
Event storage			
Alarm	Minimize e2e response time	High	1
	Maximize e2e reliability	High	1

**Table 6:** Speed profiling application constraints.

Component		Hardware	Possible Nodes
1 and 2	Telemetry	GPS	15 and 18
4 and 5	Profiler	Fast processor	1–12
6 and 7	Visualization	Dashboard	14 and 17

**Table 7:** Smoke monitoring application optimization criteria.

Path	Optimization Criteria	Priority	Weight
Train 1	Minimize e2e response time	High	1
Train 2	Minimize e2e response time	High	1

**Table 8:** Smoke monitoring deployment nodes selection.

Component		Possible Nodes	Best Case	Worst Case
1	Measurement	13	13	13
2	Batching	1–20	8	6
3	Level detection	1–20	13	10
4	Storage	1–12	8	7
5	Alarm	14	14	14
	Score		98%	43%

**Table 9:** Speed profiling deployment nodes selection.

Component		Possible Nodes	Best Case	Worst Case
1	Telemetry 1	15	15	15
2	Telemetry 2	18	18	18
3	Message broker	1–20	6	12
4	Profiler 1	1–12	5	2
5	Profiler 2	1–12	6	3
6	Visualization 1	14	14	14
7	Visualization 2	17	17	17
	Score		98%	62%
